# Correction: A genetically defined asymmetry underlies the inhibitory control of flexor–extensor locomotor movements

**DOI:** 10.7554/eLife.13038

**Published:** 2015-11-25

**Authors:** Olivier Britz, Jingming Zhang, Katja S Grossmann, Jason Dyck, Jun C Kim, Susan Dymecki, Simon Gosgnach, Martyn Goulding

Britz O, Zhang J, Grossmann KS, Dyck J, Kim JC, Dymecki S, Gosgnach S, Goulding M. 2015. A genetically defined asymmetry underlies the inhibitory control of flexor–extensor locomotor movements. *eLife* **4**:e04718. doi: 10.7554/eLife.04718

Published 14 October 2015

The originally published Figure 1 mistakenly used an image from an earlier version of the article, in which the order of the panels had been switched, resulting in a disconnect between Figure 1, the figure legend for Figure 1, and the text.

The corrected Figure 1 is shown here:

**Figure 1:**
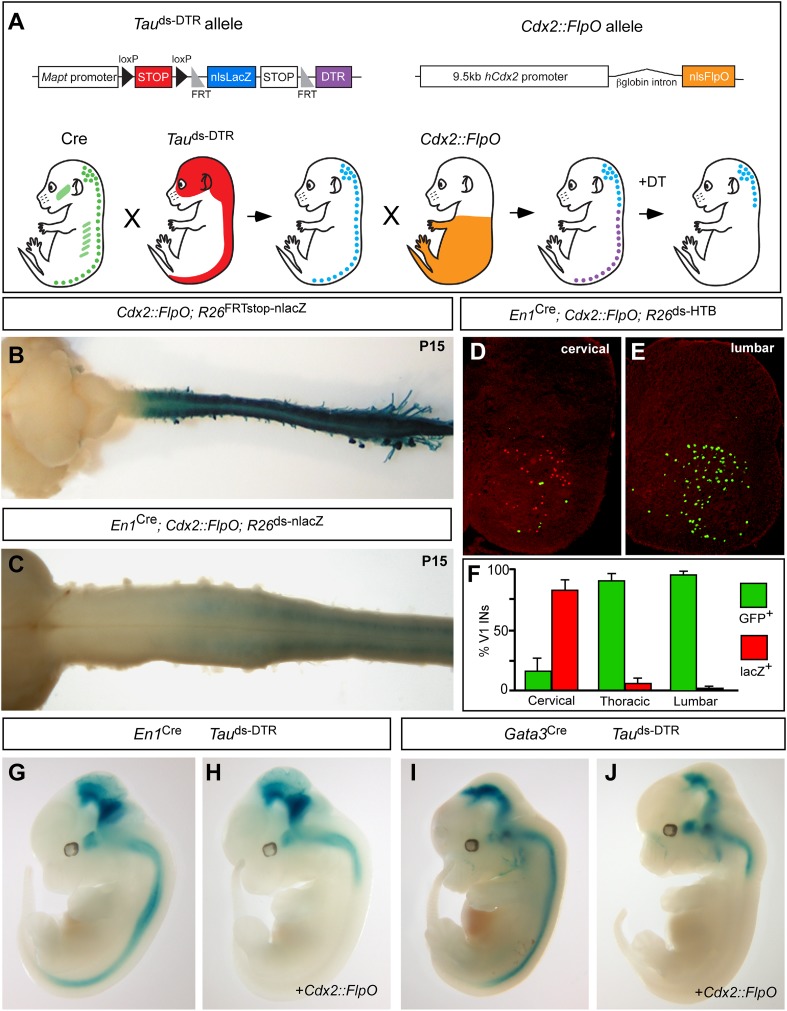

The originally published Figure 1 is also shown for reference:

**Figure 2:**
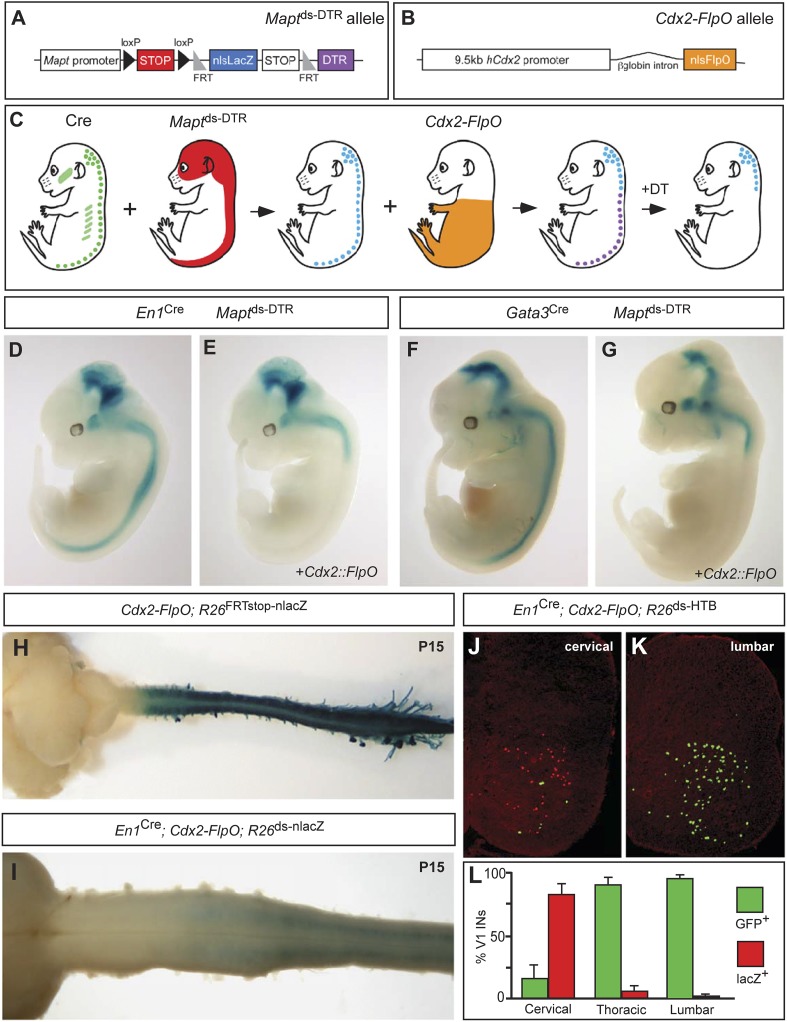

Following this change, the sentence, ‘(**J**–**L**) Sections through the spinal cord showing the relative numbers of V1 cells that undergo Cre only recombination (lacZ-positive) vs those V1 cells that undergo both Cre and Flp recombination (GFP-positive)’ has been removed from the legend of Figure 1 and the text ‘(**G**–**I**) Images from E12.5 embryos…’ has been corrected to read ‘(**G**–**J**) Images from E12.5 embryos…’

In addition, the label on the top left side of Figure 2 should have read ‘−FlpO’, rather than ‘_FlpO’.

The article has been corrected accordingly.

